# Supplementary material for: Are carbon emissions trading and green financial instruments synergistic? -Comprehensive quantitative research based on content analysis
Source: PLoS One. 2024 Mar 7;19(3):e0298601. doi: 10.1371/journal.pone.0298601 (PMC10919601; doi:10.1371/journal.pone.0298601)
Supplement: S1 Data — (ZIP) [file pone.0298601.s001.zip › Data/地级市Z_F工作报告.zip!/┴╔─■/╕o╦│╩╨/└┤╫╘╣1⁄2╓┌║┼í╛┬φ┐╦╩2╛▌═°í┐_2015.docx]

各位代表：

现在，我代表市政府向大会报告工作，请予审议，并请市政协各位委员提出意见。

一、2014年工作回顾

过去的一年，是抚顺发展史上极为艰难的一年，经济下行压力前所未有。在省委、省政府和市委的正确领导下，在市人大和市政协的监督支持下，我们团结带领全市人民，积极应对严峻复杂的经济形势，抢抓新一轮东北振兴重大机遇，全力以赴稳增长、促改革、调结构、惠民生，推动了经济社会持续稳步发展，为加快城市转型、建设幸福美丽抚顺奠定了基础。

预计完成地区生产总值1378亿元、同比增长6%，公共财政预算收入134.2亿元、增长0.03%，全社会固定资产投资921亿元、下降16%，社会消费品零售总额581亿元、增长12%，城市居民人均可支配收入25130元、增长10.2%，农村居民人均可支配收入11150元、增长11%。城乡居民收入增幅明显高于地区生产总值、公共财政预算收入增幅，城镇登记失业率、居民消费价格涨幅均在年初预控目标内。

（一）经济运行保持平稳

支持实体经济发展，免征部分小微企业增值税和营业税、减半征收小微企业所得税，6000多户企业受益；取消、减免行政事业性收费和基金，减轻企业负担9700万元；扩大“营改增”试点范围，企业减税7860万元。走进企业解决问题，抚顺石化公司、抚矿集团等重点企业中坚作用凸显，规上工业总产值增长3%、增加值增长5.5%。强化政策扶持，民营经济加快发展，实现增加值953.9亿元、增长13%。建立中小微企业综合服务平台，加大融资扶持力度，全民创业氛围浓厚。强化投资拉动，狠抓项目建设，全市开复工亿元以上重点项目373个，完成投资581亿元。

(二)抢抓机遇初见成效

大伙房水源保护区成为国家首批生态文明先行示范区；新抚老工业区率先列入东北振兴国家首批城市老工业区搬迁改造试点；新钢铁环保搬迁改造、清原抽水蓄能电站等成为国家重点推进项目；抚顺建设国家资源型城市可持续发展示范区成为国务院发展研究中心研究课题。我市被省政府确定为全省唯一旅游产业发展试点市；丰远热高乐园、抚顺文化市场成为国家级文化产业示范基地；抚顺琥珀雕刻被列入国家非物质文化遗产保护名录，“中国琥珀城”成为全省唯一入选国家“2014年度特色文化产业重点项目”；以抚顺为核心的国家级辽东文化生态保护区通过文化部审核。积极参与东北振兴央企辽宁行、全国知名民企辽宁行、沪企辽宁行活动，上海斐讯、台湾品高等一批项目成功落地；抚矿集团境外投资110亿元油页岩综合开发项目成为全省实施“走出去”战略的标志项目。

（三）城市转型启动实施

编制《抚顺市建设消费型城市中长期发展规划》、《抚顺市建设国家资源型城市可持续发展示范区顶层设计及系统发展规划》；修编《抚顺市城市总体规划》；制定推进文化创意和设计服务与相关产业融合发展行动计划、加快发展服务业的若干意见、电子商务发展规划；出台引进社会资本加强公共服务领域建设、加快发展养老服务业等实施意见，以及深入实施旅游名城战略推进城市转型的若干政策。产业结构发生积极变化，服务业增加值占地区生产总值比重提高1.5个百分点，消费对经济增长贡献率提高5.9个百分点。

（四）各项改革有序推进

完成卫计委、住建委组建和食药监局重组，公共资源交易平台逐步完善。简政放权，取消和下放行政审批权131项。抚顺铝厂等破产国企清算、职工安置工作依法推进，国有尚存竞争性企业市场化改革正式启动。完成农村土地确权登记颁证试点工作，土地承包经营权流转顺利开展。完成公汽公司、自来水公司等公用企业劳动用工及分配制度改革。完成抚顺市满族艺术剧院转企和文化市场综合执法改革。燃气价格改革稳步推进。市殡仪馆混合股份制改革进展顺利。扎实推进工商登记和企业监督管理制度改革，设立总部经济基地暨工商登记制度改革试验区，新登记内资公司制企业增长59.2%。

（五）产城一体协调发展

沈抚新城产业区加快建设，开工项目180个，完成投资328亿元；新增产值超亿元工业企业30家；太平洋工业城累计建成标准厂房110万平方米，进驻企业80家;一批项目具备生产条件;沈抚新城成为国家新型工业化产业示范基地。旅游集聚区初具规模，丰远丛林欢乐世界、冠翔冰雪大世界、辽沈战役景区建成营业，丰远热高乐园成为全国优秀旅游项目。核心区城市功能不断完善，多家银行机构、小贷公司、担保公司入驻，亿丰家居建材村一期百家工厂店试营业，迪卡侬品牌运动超市、大商新玛特购物中心开业。

石化新城化工及精细化工产业基地进驻企业突破100家、建成投产62家，引进世界500强霍尼韦尔催化剂、营口港物流等超亿元项目9个，同益石化、齐隆化工等骨干企业平稳运行。服务业日益繁荣，兴隆大家庭生活购物广场开业，大商新玛特、中惠时代广场主体建成，北京广联达汽车4S店8个品牌店投入使用。城市功能不断完善，完成搭连立交桥等10项城市基础设施建设，甲邦大桥竣工，河滩公园建成使用。

（六）结构调整步伐加快

工业结构优化升级。开工亿元以上重点项目195个，完成投资350亿元。全社会研发投入增长21%，新增国家级高新技术企业9家，完成高新技术产品增加值208亿元、增长10%。全市重点工业产业集群实现销售收入1910亿元、增长16.7%。隆基电磁等4户企业引进海外先进技术，新钢铁、红透山铜矿、天湖啤酒等企业信息化和工业化融合工作走在全省前列。

服务业异军突起。支持电商发展，沈抚新城电子商务中心正式启动、引进企业50余家，“智慧电商谷”文化电商产业园、同城物联大厦、东北网贸港相继建成，全市电子商务交易额增长40%。加快集聚区建设，新抚中央商务区主营业务收入进入全省10强，新宾满族文化旅游休闲集聚区晋升省级现代服务业集聚区。强化项目支撑，中国供销集团东北商贸城、兴隆摩尔世界等项目进展顺利，沈抚新城出版印刷创意产业园建成并投入使用。推进工业企业主辅分立，57户企业分立出生产性服务企业68户，超额完成省政府下达任务。大力实施旅游名城战略，新建完善旅游项目11个，推出五大系列旅游产品，开通青岛、南昌等省外23条旅游专列，首开“引客入辽”先河，抚顺建设旅游目的地城市初见成效，在全省旅游业发展综合排名连续两年第一。

金融业快速发展。创新融资模式，三宝屯污水处理厂成为全省特许经营权成功转让第一家企业，被财政部列为政府和社会资本合作示范项目；积极运用小微企业增信集合债、融资租赁、定向债务融资，获批融资额度51亿元，为历史最好水平。积极利用资本市场，32家企业在辽宁股权交易市场挂牌、数量在全省排名第三，抚顺县天利机铸等4家企业在上海股权托管交易中心Q板挂牌。金融机构发展壮大，新批小额贷款公司29家、融资担保公司3家，新增证券公司4家，辽宁国拓成为首家落户抚顺的投资基金管理公司。金融对全市经济支持作用增强，全年新增贷款76亿元，新增存贷比增长91.5%；非信贷融资164亿元，实现翻番增长；金融业税收增长65%。

现代农业发展壮大。有效应对63年来最严重旱灾，粮食产量实现年初目标。调优产业结构，以大伙房水源保护区综合治理为契机，完成有机农业栽种面积7.3万亩；创建国家级万亩粮油高产示范区14个，农业标准化生产面积达到168万亩；食用菌、中药材、山野菜、花卉四大特色产业基地规模不断壮大，全省最大集中连片食用菌种植基地落户抚顺；大力发展生态畜牧业，实现畜牧业总产值57.4亿元、增长5.4%。全年无重大农产品质量安全事故。

（七）城乡面貌持续改观

生态保护力度加大。深入推进大伙房水源保护区综合治理，完成88个治理项目评审和保护区勘界、一级保护区退耕工作，生态移民、畜禽养殖场搬迁等扎实推进。全面治理大气污染，完成60个蓝天工程项目，拆除135台10吨以下燃煤锅炉，竣工36个工业提标改造项目，新开10条汽车尾气检测线，关停170户龚家地板城环保不达标企业，城市空气质量综合指数全省排名比上年前移四位。稳步推进青山工程，完成荒山造林10.2万亩、经济林带12.7万亩、闭坑矿生态治理1377亩。城区种植乔灌木203万株，绿化美化硬化84个居民小区。全面推行无公害除雪。

城市功能日臻完善。启动辽宁中部环线抚顺段高速公路建设，完成青台子街等8条道路改造，居民入户街巷路改造工程顺利推进。新建改造“三供”管网159.8公里，建成投用演武生活垃圾处理厂和3座污水处理厂，城市生活垃圾实现无害化达标处理，污水处理能力达到100%。投资6.7亿元，更新新能源公交车852台，新建改造公交站亭267个、站牌900个，调整延伸公交线路43条次，新建改造公交场站、司乘人员休息室和配餐中心，改善了司乘人员工作生活环境，基本解决了百姓出行难问题。新华桥重建当年通车，城市南北通行能力显著提升。实施“圈抚顺”工程，结束了抚顺没有动车的历史。

美丽乡村成效初显。全面推进农村环境综合治理，维修县级以上公路400公里，改造农村公路160公里，建设“一事一议”村内道路345公里。完成绿化示范村125个，农村安装路灯4500盏，185个行政村环境明显改善。新宾成为“2014中国最美丽县”全省唯一上榜县、赫图阿拉村成为首批中国少数民族特色新村，红透山、后安等5个乡镇成为全省宜居示范乡镇，抚顺县成为全省亮化工程试点县。

（八）民生保障扎实有力

社会保障水平不断提升。克服重重困难，企业离退休人员养老金按时足额发放。调增企业退休人员养老金、城乡居民基本养老保险基础养老金、城乡低保和农村五保供养标准，提高城镇居民基本医疗保险和新农合政府补助标准，实施居民大病保险和农村住房灾害保险。创新互助式扶贫开发模式，整村搬迁移民扶贫475户，减少农村贫困人口4.4万人。完成市社会福利院、市养老院维修改造工程。医疗救助4.8万人次，“精神康宁”项目救助城乡低保和低保边缘户重性精神障碍患者364人次。开展农村分散供养五保老人呼叫服务救助活动。投放妇女小额担保贷款6.8亿元。新建和改扩建城市社区办公用房81个，社区办公经费由每年2万元提高到3万元。

就业安居工作扎实推进。实施更加积极的就业政策，促进高校毕业生和困难群众就业，实名制就业9.5万人，零就业家庭动态为零。棚改新开工30.5万平方米、基本建成52万平方米，公租房和廉租房配户3053户，近6500户采沉棚户区居民避险搬迁安置工程主体完工，完成“暖房子”工程22万平方米，改造农村危房530户。解决了2.3万户市民吃水难、用气难问题，建设农村安全饮水工程116处、惠及群众4.2万人。积极解决万达西、大酒店、卧龙湾等拆迁遗留问题和商品房产权证办理难问题。

各项社会事业协调发展。望花区、顺城区通过国家义务教育发展基本均衡县验收，义务教育阶段学校新生入学实行“阳光分班”，省示范性高中招生实行平行志愿。市妇幼保健院改造、市中心医院病房楼、市第二医院儿科基地项目开工建设，市肿瘤医院续建项目竣工，医患纠纷第三方调解机制建立，清原县被评为全国计划生育优质服务先进县。成功举办“群众文化节”和“读书节”，开展各类群众文化主题活动2200场，市图书馆竣工开馆，抚顺琥珀列入国家地理标志保护目录。成功举办东三省首届高尔山山地自行车邀请赛和辽宁省首届三块石国际登山系列活动，成功承办第五届全国大学生龙舟锦标赛。残疾人综合服务中心开工建设，老年、妇女儿童活动中心和青少年宫投入使用。

社会管理能力逐步提高。刑事案件发案率下降22.3%，信访总量下降17.5%，安全生产和食品药品安全形势基本平稳，事故救援、抗灾减灾能力进一步增强，气象灾害预警信息实现村屯全覆盖。

在社会各界大力支持下，完成“8·16”灾后恢复重建，南口前新市镇集中安置房达到入住条件，水毁道路、桥梁、堤防等重建工程均达到或超过灾前水平。

（九）自身建设不断加强

坚持问题导向，认真整改落实。深入开展党的群众路线教育实践活动，聚焦“四风”问题，整改群众关心的热点难点问题386个、政府党组及班子问题118个，政府更具凝聚力和向心力。

转变会风文风，为民务实守信。严格执行中央八项规定，全市政府性会议减少25%、文件减少21%，压缩“三公”经费19%，集中财力投向民生。工作有部署、有督查、有结果，政府执行力、公信力增强。

严格依法行政，自觉接受监督。认真执行市人大及其常委会的决议决定，自觉接受人大、政协监督，主动听取社会各界意见建议。全年办理建议393件、提案423件，办复率100%，解决率67%；受理市长公开电话3万件次，转办1.65万件次,群众满意率98.8%。

其它各项工作都取得了积极进展。

各位代表，过去的一年，我们变压力为动力，抓机遇促发展，用开拓进取、务实苦干，赢得了来之不易的发展成果。这是省委、省政府和市委正确领导的结果，是市人大、市政协监督支持的结果，是全市人民团结奋斗和社会各界无私帮助的结果。在此，我代表市政府，向全市各族人民，向人大代表、政协委员，向各民主党派、工商联、无党派人士、人民团体、离退休老同志，向驻抚部队、武警官兵、公安干警和中省直单位，向所有关心抚顺发展的朋友们，表示崇高的敬意和衷心的感谢！

看到成绩的同时，问题不容回避。抚顺老工业基地长期积淀的矛盾还没有得到根本解决，加快转型振兴的任务依然艰巨。一是市场有效需求不足，传统骨干企业生产运营困难，民营经济竞争力不强，小微企业发展缓慢，确保经济平稳增长压力较大。二是项目引进和储备不足，投资拉动作用不强，经济增长后劲乏力。三是群众普遍关注的大气污染、食品药品安全、冬季供暖、教育医疗就业等问题，还没有妥善解决。四是部分公务人员对经济发展新常态认识不足，不敢作为，不会作为，主动适应新常态的能力有待提高。我们将直面问题，采取有力措施积极应对、认真解决。

二、2015年主要工作

今年政府工作的总体要求是：以党的十八大和十八届三中、四中全会精神为指导，全面贯彻落实省、市委全会暨经济工作会议精神，坚持稳中求进工作总基调，主动适应经济发展新常态，紧紧抓住新一轮东北振兴重大历史机遇，深入实施“两城两带一区、多点支撑、全域发展”战略，以城市转型为主线，以项目建设为核心，以经济运行为重点，着力稳增长、促改革、调结构、惠民生，加快建设幸福美丽抚顺。

全市经济社会发展的预期目标是：地区生产总值增长6%左右，公共财政预算收入增长2%左右，全社会固定资产投资增长6%左右，社会消费品零售总额增长11%左右，城乡居民人均可支配收入分别增长10%左右。

为实现上述目标，着力抓好以下八项工作：

(一)积极应对挑战，确保经济平稳

支持企业发展。继续走进企业，坚定不移为企业减负，支持企业做强做大。强化领导包保，建立“问题清单”，“一企一策”解决问题，确保现有企业正常运营、满负荷生产。强化政策扶持，加强协作配套，健全融资担保机制，着力解决“融资难”、“融资贵”问题，支持小微企业发展，形成大众创业、万众创新的崭新局面。

抢抓历史机遇。紧紧围绕《国务院关于近期支持东北振兴若干重大政策举措的意见》，谋划推出一批具有全局性、战略性重大项目，争取国家新的支持。推进争取到的国家支持项目尽快开工、早日见效。密切跟踪国家政策资金支持方向，在矿山地质灾害综合治理、资源枯竭型城市可持续发展等方面，争取先行先试，为项目建设创造条件。

继续扩大开放。鼓励外向型经济发展，争取国家将罕王集团投资印尼镍矿产业园项目,纳入“一带一路”战略重点建设项目，支持抚顺县救兵保税仓库等项目建设。深入挖掘产业、资源和区位优势，策划包装一批产业升级、城市转型等重点项目。创新招商方式，优化投资环境，继续“走出去”、“请进来”，力争在引进战略投资者、建设牵动性大项目上实现新突破。

（二）坚持改革创新，增强发展活力

以更加坚定的信心深化改革。以沈抚新城行政审批制度改革试点为突破口，加大全市行政审批制度改革力度，大幅压缩审批事项，切实提高审批效率。完善政府投融资管理体制，吸引民间资本进入公共服务领域。加快国资国企改革，推进市属国有公益性骨干企业建立现代企业制度。继续深化公共资源交易管理体制和商事登记制度改革。推进财税体制改革、预算公开透明，加强政府债务管理和风险防控。建立农村产权交易市场，加快土地流转，引导和规范集体经营性建设用地入市，完善农村宅基地管理和土地征收补偿等制度。加强高层对接，力争沈抚同城取得实质性进展。

以更加有力的举措推动创新。坚持创新驱动，重点推进石化新城化工及精细化工、沈抚新城机器人与智能装备制造、新宾焊接材料及装备等技术创新联盟建设，支持油研、煤研国家级实验室建设，争取“沈-大”国家自主创新示范区政策辐射两座新城。实施“高新技术企业百家工程”，培育创新型企业30家、知识产权优势企业10家，高新技术产品增加值增长10%。

(三)优化产业结构，推进城市转型

加快工业转型升级。提升改造传统产业，推进信息化与工业化深度融合，鼓励支持煤、油、钢、铝等重点骨干企业加大创新力度，搞好精深加工，拉长产业链，实现传统优势产业高端化、产品终端化。加快发展新兴产业，把握国家产业政策，加强与国内一流科研院所合作，加快发展高端装备制造、通用航空和新材料等战略性新兴产业，培育新的经济增长点。做大工业产业集群，引导吸纳社会资本参与园区基础设施建设，加快大型龙头企业和上下游配套产业向园区集聚，全市重点工业产业集群实现销售收入2100亿元、增长10%。

促进服务业发展提速。加快亿丰电子商务交易中心和东北网贸港网商基地建设，争取抚顺成为国家电子商务示范城市、沈抚新城成为全省电子商务产业基地；加快金融、会展、总部经济等现代服务业发展，努力创建层次高、业态新、辐射强的现代服务业集聚区。加快森茂国际汽车城、泰为汽车文化城、亿丰东北家居建材品牌中心、东北亚木业交易中心建设，确保印象新城、兴隆摩尔世界等大型商业综合体投入运营，打造一批高品质、国际化的现代物流园区。深入推进工业企业主辅分立，加快发展生产性服务业。推动琥珀煤精产业发展，创建中国珠宝玉石特色产业基地。深入实施旅游名城战略，加快建设丰远温泉养生世界、岗山林溪养生福地、大伙房国家湿地公园和皇顶山森林公园等重点项目；整合旅游资源，加强互联互通，打造十大最美乡村、十大风情小镇、十大养生基地，加快建设旅游目的地城市，全年旅游总收入增长20%。坚持多措并举，积极促进房地产业健康发展。

做大特色农业规模。转变农业发展方式，加大科技兴农力度，打造特色化、优质化和品牌化的现代农业体系。以大伙房水源保护区成为国家首批生态文明示范区为契机，建成有机、绿色农业示范基地17.6万亩。做大食用菌、中药材、山野菜和花卉四大特色产业基地规模，扶持辉山乳业等重点农产品深加工项目，以龙头连农户、带基地、接市场、创品牌，带动农民增收致富。大力推进传统农业与食品加工、旅游休闲、健康养生等产业融合发展，提升全产业链竞争力。继续加大农业投入，完善农业基础设施，加强农机推广，提高农业抗灾减灾能力。

(四)加快区域发展，强化多点支撑

沈抚新城，用足沈阳经济区支持政策，加快机器人、现代印刷和电子商务特色产业基地建设，重点推进陌越智能产业园、中联智鼎创意大厦、海油金融大厦等项目。依托旅游、商贸和金融等服务业集聚区，加快卓华欧洲名品一条街和沈抚新城金融街等项目建设。完善道路、管网等城市基础设施，启动沈抚综合客运枢纽站建设，推进沈抚地铁直通，提升公共服务水平，吸引人口集聚。

石化新城，加快与抚顺石化公司共建产业园区步伐，做强做大化工及精细化工产业基地；完善基础配套设施，加快石化新城热电厂建设；重点推进霍尼韦尔催化剂、伊科思液体炼胶、三木集团丙烯酸树脂等项目，确保佳化化学等企业达产达效。加快老城区更新改造进程，启动新屯、章党地区路改工程，规划建设临江路、新城路东延等项目，形成南北贯通的主要路径构架，构建与城东地区连片开发的新格局。

南环产业带，重点加快望花经济开发区和胜利经济开发区建设。望花经济开发区围绕110公顷起步区，加快园区基础配套设施建设，推进恒德磁业、电线电缆产业园等重点项目；胜利经济开发区围绕南环中部产业集群核心区，启动再生资源产业园一期等项目，推进食品工业园达产达效。

浑河景观带，继续提升环境、完善功能，依托好运角、人民广场、万达广场、月牙岛和金风湾等重要节点，发展商贸服务业，带动周边区域开发，推动人居向浑河两岸集聚。

东部生态区，依托大伙房水源保护区综合治理和辽东文化生态保护区建设，加快“三县一区”产业结构调整步伐。因地制宜，大力发展有机、绿色农业，创出抚顺特色品牌；立足优势，大力发展沟域旅游、乡村旅游和健康服务业，推动服务业提质增效；着眼长远，大力发展低碳、环保、高效产业，推动工业转型升级。

(五)狠抓项目建设，增强发展后劲

强化要素保障。积极争取建设用地指标，用足用活工矿废弃地复垦和增减挂钩政策，确保有限的建设用地向成熟项目和重点项目倾斜。拓展融资渠道，培育金融资本市场，支持企业上市，发展各类投资基金，鼓励引导民间资本参与重大基础设施和公用工程建设。完善鼓励政策，支持小额贷款公司、融资担保公司和地方银行加快发展。全年新增贷款90亿元，非信贷融资160亿元。

完善推进机制。严格落实重点项目领导包保责任制和重大项目协调推进机制，建立软环境责任追究制度，做好立项审批、投资评审、开工建设、竣工投用等全过程保障服务工作，着力解决影响项目引进、建设和达效的突出问题，让项目引得进、留得住、发展得好。

突出重点项目。加快推进上海斐讯、中文时代等新建项目，支持煤研产业基地、抚特钢精品钢材基地等续建项目，推动抚矿热电厂等在建项目投产达效。全年开工建设亿元以上重点项目236个、总投资2215亿元，当年完成投资435亿元。

(六)全面改善环境，建设宜居城市

加强生态保护。贯彻落实《辽宁省大伙房饮用水水源保护条例》，深入推进大伙房水源保护区综合治理和国家江河湖泊生态保护重点项目建设，扎实开展一级保护区生态移民、畜禽养殖业污染治理等重点工作，启动水库上游河道生态带建设，建成投用20个乡镇污水处理设施。综合整治大气污染，继续推进龚家地板城环保搬迁改造，按期完成重点行业污染治理设施提标改造，加大对造污企业、小作坊治理和重污染企业监管力度，控制扬尘污染和化工异味，减少雾霾天气。实施浑河干支流综合整治，确保企业污水达标排放，城市生活垃圾及渗滤液无害化处理率100%。继续实施青山工程，完成荒山造林5.5万亩、封山育林30万亩、闭坑矿生态治理1577亩。

加快城乡建设。积极推进国家资源型城市可持续发展示范区起步区建设，开工建设生态运动廊道、示范区规划展示馆、“东北记忆”工业文明主题广场，推进中国北方越野车运动公园等项目建设。以新抚老工业区搬迁改造为契机，建设工业遗址生态公园和琥珀文化产业园，推进永济路和千金路沿线工业企业搬迁改造、华山地区综合开发建设。推进城东三期基础设施建设。加快城市街心小游园、小广场、小绿地和雕塑建设，提升公园、高速公路出口、高山路沿线、大伙房水库周边重要节点景观。完善“圈抚顺”铁路配套工程，加快辽宁中部环线抚顺段高速公路建设，大中修县级以上公路215公里。推进居民入户街巷路改造工程，加大城市供暖、供水和燃气管网改造力度。以人为本，遵循规律，积极稳妥推进新型城镇化。深入推进农村环境综合治理，重点抓好农村垃圾分类处理、畜禽粪便集中处理和新型能源建设,重点整治行政村159个、集中绿化村屯100个、维修改造农村公路197公里，推动美丽乡村建设再上新台阶。

注重城市管理。将城市管理纳入绩效考核体系，接受媒体和社会监督，调动城区积极性，构建“大城管”格局。从群众反映较为强烈的突出问题入手，继续加大站前地区交通秩序综合整治力度，着力解决乱挖道路、违章建筑、占道经营、乱停乱放等城市管理顽疾。调整完善公交线网和站点布局，规范交通旅游等城市导览导视标识，全面推行金融IC卡应用，继续推行无公害除雪。积极倡导文明祭祀。加快“智慧城市”建设，建设城市地下管网、综合信息管理和基础地理信息共享平台，尽快实现地下管网数字化、智能化管理。

环境是生存之基、发展之本，青山绿水是最宝贵的财富。我们一定要保护好抚顺的山山水水，让蓝天永驻、碧水长流，让我们的家乡更美丽、城市更宜居。

(七)全力保障民生，构建和谐社会

提高社会保障水平。强化扩面征缴，加大争取力度，确保企业离退休人员养老金按时足额发放。继续提高城镇居民医疗保险和新农合政府补助标准，做好异地居住人员医疗保险即时结算试点工作。提高城乡低保救助标准，完善低保困难家庭学生救助政策，实施“精神康宁”等慈善救助项目。加大扶贫帮困力度，整村搬迁集中安置移民扶贫300户、科技到户扶贫1100户，组织贫困劳动力培训4300人。

完善公共服务体系。办好人民满意教育，支持各级各类教育发展，打造一流的校长和教师队伍，提升教育教学质量。取消政府办县、乡公立医疗机构及一体化村卫生室药品加成，建成投用市中心医院病房楼和第二医院儿科基地，完成市妇幼保健院改造工程。推进文化惠民，建成社区书屋139家，组织群众性文化活动2200场。加强村（社区）体育设施建设，新增健身路径和体育设施230处，开展各级各类群众性体育活动100项。加大公办养老服务设施建设力度，支持鼓励民办养老机构发展。深化拥军优属工作，适时提高优抚对象抚恤标准，积极创建双拥模范城。新建和改扩建社区办公用房35个。

实施就业安居工程。多渠道扩大就业，回抚应届高校毕业生就业率力争达到96%以上，全年完成实名制就业7.5万人，城镇登记失业率控制在5%以内。全方位保障安居，新增棚改安置房1.5万套，改造农村危房610户，实施“暖房子”工程20万平方米以上；加强老旧、弃管小区物业管理，妥善解决集中连片棚改房屋维修和小区修缮问题；新建改造民用燃气管网20公里，建设农村安全饮水工程174处、惠及群众8.4万人。

确保社会和谐稳定。加大曝光打击力度，严格落实“黑名单”制度，对食品药品安全问题，发现一起、查处一起，决不手软、决不姑息。逐步推进法律服务重心下沉，每个县区建立1个村（社区）综合法律服务室。继续强化社会治安综合治理，提高群众安全指数。认真对待群众合理诉求，着力化解一批影响较大的信访积案。建立“党政同责、一岗双责、齐抓共管”的安全生产责任体系，减少较大事故、杜绝重特大事故。切实维护民族团结。做好气象灾害预防、应急处置工作。

继续做好其它各项工作。

各位代表，越是困难时期，我们越要高度关注民生、全力保障民生，该花的钱一分不少，该做的事一定做好，努力让全市人民的日子越过越红火、生活越来越幸福。

(八)坚持依法行政,建设满意政府

建设法治政府。深入贯彻十八届四中全会精神，坚持法无授权不可为、法定职责必须为，不断规范行政权力运行，坚决杜绝选择性执法、多头执法、滥用行政裁量权等行为，全面提高依法行政水平。

建设诚信政府。重信守诺，凡是政府出台的政策措施，凡是政府对企业、群众和投资者作出的承诺，坚决落实，严格兑现，以诚信政府建设带动诚信社会建设。

建设服务政府。主动适应经济发展新常态，善于学习、勇于担当、敢于作为，千方百计为企业、群众和投资者提供优质、高效服务，让企业有发展、群众得实惠、投资者真满意。

建设廉洁政府。严格遵守党风廉政建设各项规定，以身作则、廉洁自律，强化审计监督和行政监察，全力整治“吃拿卡要、庸懒散拖”问题，以良好的党风政风带动社会风气转变。

各位代表，抚顺已经踏上全面深化改革、转型振兴发展的伟大征程。任何困难都改变不了抚顺人民追求幸福生活的美好愿望，任何挑战都阻挡不了抚顺人民加快转型振兴的奋进步伐。让我们在市委的正确领导下，解放思想，开拓进取，奋发有为，共同谱写城市转型、幸福美丽抚顺建设新篇章！
